# Acinar cells in the neonatal pancreas grow by self-duplication and not by neogenesis from duct cells

**DOI:** 10.1038/s41598-017-12721-9

**Published:** 2017-10-03

**Authors:** Isabelle Houbracken, Luc Bouwens

**Affiliations:** Cell Differentiation Lab, Faculty of Medicine and Pharmacy, Vrije Universiteit Brussel, Brussels, Belgium

## Abstract

Pancreatic acinar cells secrete digestive enzymes necessary for nutrient digestion in the intestine. They are considered the initiating cell type of pancreatic cancer and are endowed with differentiation plasticity that has been harnessed to regenerate endocrine beta cells. However, there is still uncertainty about the mechanisms of acinar cell formation during the dynamic period of early postnatal development. To unravel cellular contributions in the exocrine acinar development we studied two reporter mouse strains to trace the fate of acinar and duct cells during the first 4 weeks of life. In the acinar reporter mice, the labelling index of acinar cells remained unchanged during the neonatal pancreas growth period, evidencing that acinar cells are formed by self-duplication. In line with this, duct cell tracing did not show significant increase in acinar cell labelling, excluding duct-to-acinar cell contribution during neonatal development. Immunohistochemical analysis confirms massive levels of acinar cell proliferation in this early period of life. Further, also increase in acinar cell size contributes to the growth of pancreatic mass.We conclude that the growth of acinar cells during physiological neonatal pancreas development is by self-duplication (and hypertrophy) rather than neogenesis from progenitor cells as was suggested before.

## Introduction

Pancreas tissue consists of exocrine acinar and duct cells, and of endocrine cells dispersed in the islets of Langerhans. By far the majority of the volume of the pancreas consists of exocrine acinar cells. They synthesize large amounts of zymogens and digestive enzymes, which are secreted into the ductal tree leading to the duodenum. The pancreatic endocrine part makes up only 1–2% of pancreatic tissue. During embryonic development of the pancreas, all these epithelial cell types originate from a common pool of multipotent endoderm-derived progenitor cells. However, this multilineage potential progressively becomes restricted when the multipotent progenitor cells become organized into tip and trunk regions, starting at around embryonic day E12.5. The trunk domains will eventually give rise to the islet and ductal lineage, and the tip domains to the acinar lineage^1,2^. Still some dispute exists as to whether multipotent progenitors might remain present in postnatal pancreatic tissue and whether they might contribute to tissue homeostasis or repair. Alternatively, the differentiated pancreatic cells may retain sufficient plasticity to self-proliferate and maintain or increase their numbers.

Historically, studies on pancreas development and growth have mainly concentrated on the endocrine part of the pancreas, to aid in finding new treatments for diabetes. However, progressively more research is conducted concentrating on the exocrine pancreas development and growth. This is because accumulating evidence is emphasizing the role of exocrine acinar cells in pancreas pathologies such as pancreas cancer but also because the remarkable acinar plasticity might be used to generate more beta cells as a treatment for diabetes.

Diabetes results from defects in insulin secretion, or action, or both^3^. Diabetes is a growing public health problem with 1 in 11 adults (415 million) having diabetes, and with projections for 2040 of 642 million adult patients^4^. Beta cell therapy to restore the beta cell mass in diabetes patients by transplantation of islet cells is a hopeful treatment. Nevertheless, the major hurdle to overcome for large-scale beta cell therapy remains severe donor shortage. Therefore, in order to regenerate a functional beta cell mass, researchers suggested several cell types as an alternative source to generate new beta cells, including acinar cells^5–13^.

Pancreas cancer is another pancreas pathology of great concern. Exocrine tumours are the most common form of pancreas cancer with more than 85% being pancreatic ductal adenocarcinoma (PDAC). Plenty of studies have demonstrated that PDAC and PanIn arise from acinar cells^14–23^. Thereby, acinar cells undergo acinar-to-ductal metaplasia.

There are still gaps in our understanding of the normal exocrine tissue growth and renewal in the postnatal pancreatic organ. This is best addressed by genetic lineage tracing. The initial ElastaseCreERT tracing studies demonstrated regeneration of acinar cells after pancreatitis and partial pancreatectomy by acinar cell replication. However, physiological postnatal pancreas growth was not studied^24,25^. Two duct-tracing studies suggested a substantial contribution of duct cells to acinar cells postnatally with up to 85% of reporter positive cells being acinar^26,27^. Two other duct-tracing studies contradicted this with no evidence for a duct-to-acinar cell contribution in neonatal and adult mice^28,29^. The latter were confirmed by an acinar tracing study using Ptf1aCreERT mice^11^. This study showed no decrease in labelled acinar cells between 5 weeks and 7 months of age indicating that acinar cells self-duplicate to maintain the adult acinar pool. Unfortunately, these conclusions could not be drawn for the neonatal period as data on acinar labelling shortly after the pulse was lacking^11^.

In retrospect, relatively few studies have addressed the neonatal period by lineage tracing although this represents a major dynamic period with an important expansion of both exocrine and endocrine pancreas and with clear indications of higher plasticity compared to adults^30^. Here, we employed 2 different transgenic mouse strains to study cellular contributions in the exocrine acinar development during this neonatal period.

## Results

### Physiological growth in neonates

**Figure 1:**
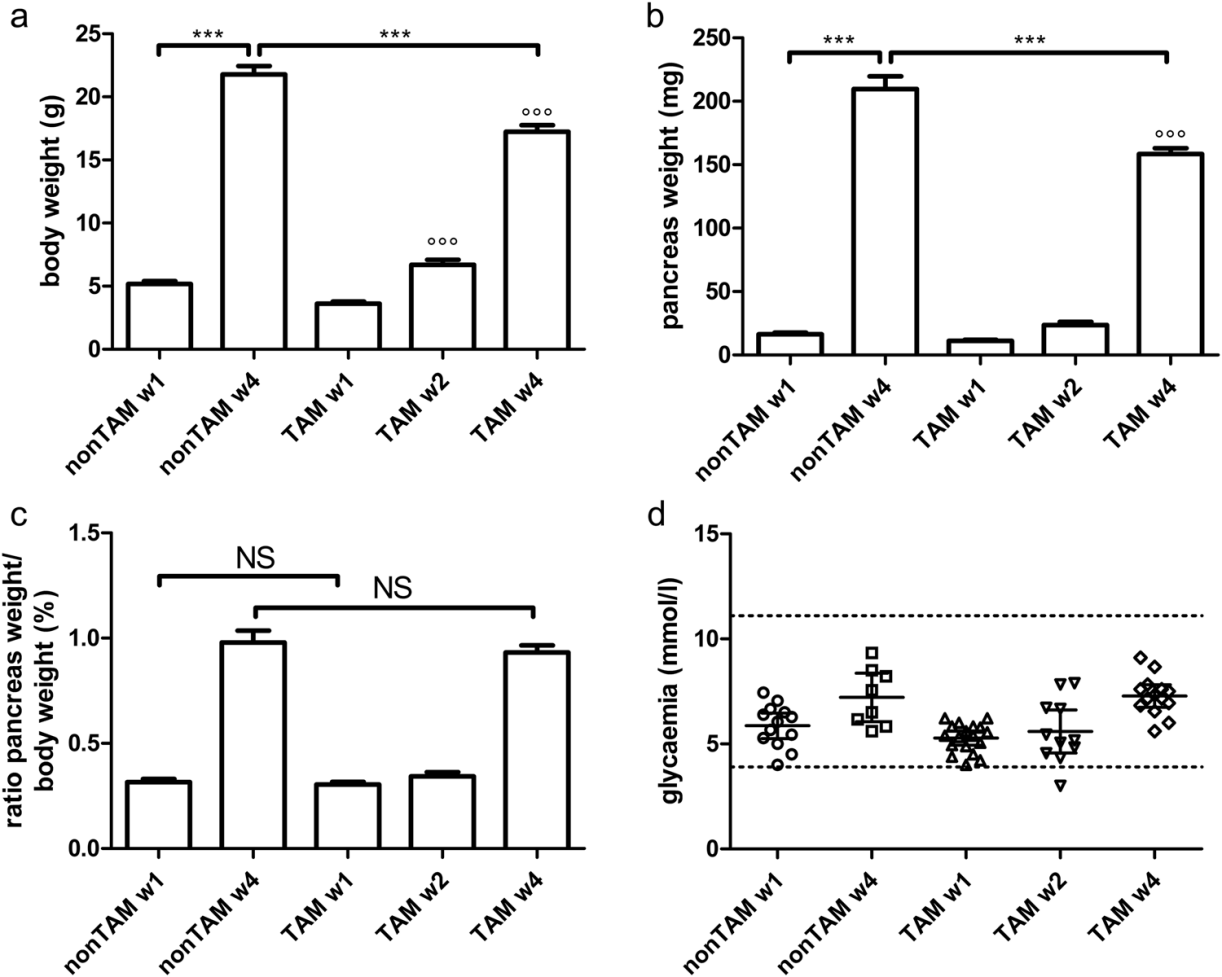
Follow-up of ElaCreERT R26-YFP mice during early postnatal period. Body weight (**a**), pancreas weight (**b**), ratio of pancreas weight over body weight (expressed as percentage) (**c**) and non-fasting glycaemia (**d**) of ElaCreERT R26-YFP mice were measured at indicated time points in non-TAM-treated mice (n = 8–14) and TAM-treated mice (n = 11–23). Data on glycaemia are expressed as mean ± 95% confidence intervals. All other results are expressed as mean ± SEM. (**a**–**c**) were analysed by 1-way ANOVA, followed by Bonferroni’s multiple comparison test (comparisons of interest: w1-w2, w2-w4, w1-w4; n1-w1, n4-w4, n1-n4. For Fig. 1c: n1-w1, n4-w4). Results are considered statistically significant when P < 0.05. *** or °°° P < 0.001. (°°°) indicates P-value compared to all previous time points. NS: not significant. Detailed information about numbers of analysed mice, cells and area can be found in Table 1.

To study the neonatal development of the exocrine pancreas we used a Cre-Lox-based tamoxifen (TAM)-inducible lineage tracing approach driven by the elastase-promoter. The physiological development of ElaCreERT R26-YFP mice was followed during the first 4 weeks of life. The body weight rises sharply during the first few weeks of life in both TAM- and non-TAM-treated mice (Fig. 1a). We observed a 4- to 6- fold increase in body weight between 1-week and 4-weeks of age in non-TAM-treated and TAM-treated animals, respectively. The relative growth of the pancreas is even higher, namely 13-fold in non-TAM-treated and 14-fold in TAM-treated mice (Fig. 1b). It is of note that both pancreas and body weight are significantly lower at P28 in TAM-treated compared to non-TAM-treated animals, indicating that TAM injection at birth, which is necessary for lineage tracing, negatively influences weight gain during neonatal development (Fig. 1a,b). However, we can exclude a selective (negative) effect of TAM injection on pancreas growth since no difference is observed in the ratio of pancreas weight over body weight between TAM- and non-TAM-treated mice neither at 1-week of age nor at 4-weeks of age (Fig. 1c). Non-fasting glycaemia remained normal throughout this time period (Fig. 1d).

### Neonatal acinar tissue expands by acinar cell proliferation and hypertrophy

**Figure 2:**
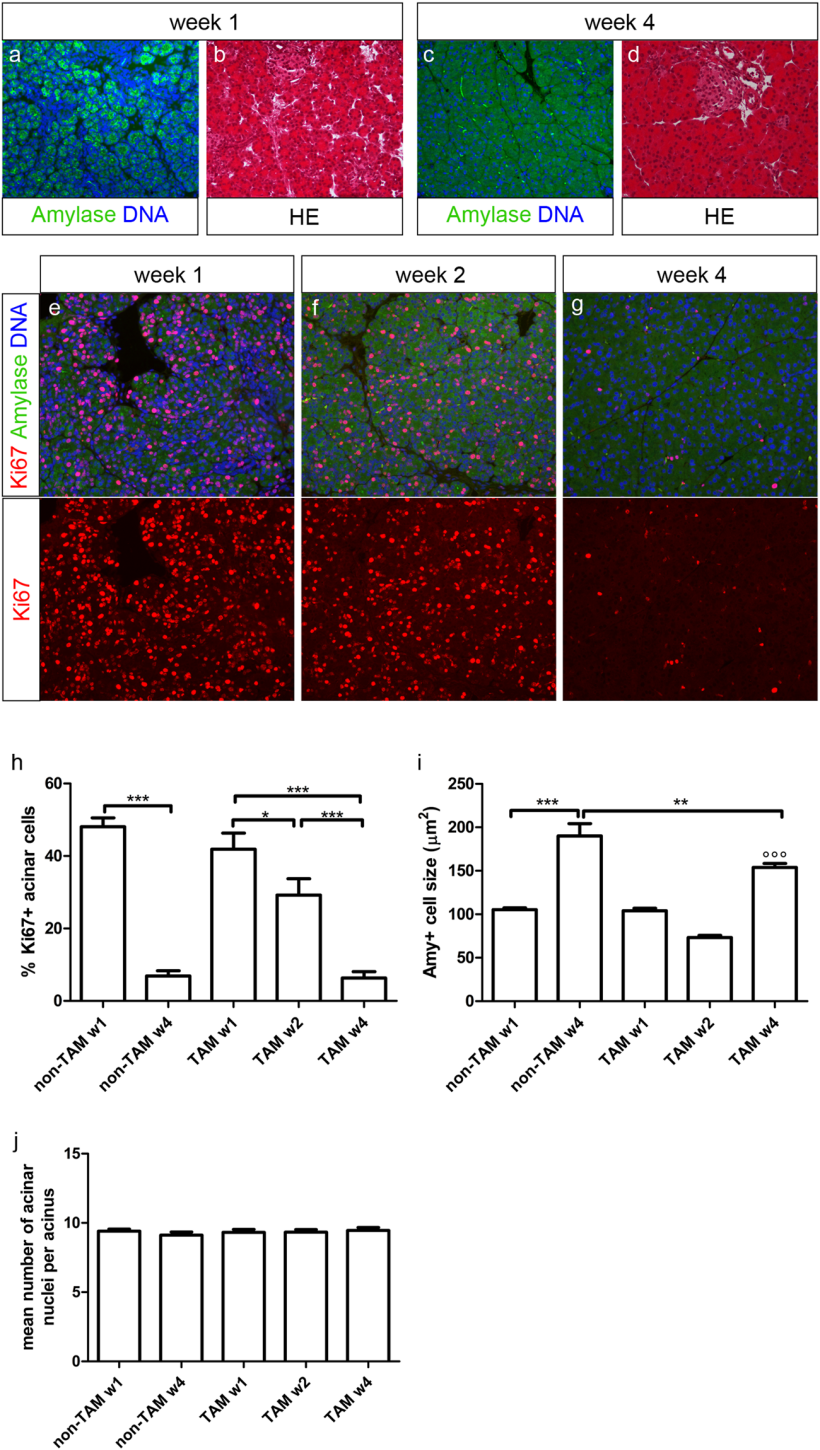
Expansion of neonatal acinar tissue by proliferation and hypertrophy. Immunohistochemical staining for amylase (**a**,**c**) and haematoxylin-eosin staining (**b**,**d**) on pancreas from ElaCreERT R26-YFP mice at 1-week (**a**,**b**) and 4-weeks (**c**,**d**) of age shows differential pancreas morphology during neonatal development. Analysis of acinar proliferation by immunohistochemical staining for amylase and Ki67 was performed at different time points (**e**–**h**). Acinar cell size (**i**) and cellularity of acini (**j**) at indicated time points. Shown are representative photomicrographs at original magnification of ×20. Results are expressed as mean ± SEM. All data were analysed by 1-way ANOVA, followed by Bonferroni’s multiple comparison test (comparisons of interest: w1-w2, w2-w4, w1-w4; n1-w1, n4-w4, n1-n4). Results are considered statistically significant when P < 0.05. *: P < 0.05, **: P < 0.01, *** or °°°: P < 0.001. (°°°) indicates P-value compared to all previous time points. Detailed information about numbers of analysed mice, cells and acini can be found in Table 1.

The pancreas morphology undergoes significant alterations during neonatal development. Initially, the acinar tissue is compact and dense at 1 week of age (Fig. 2a,b). But by week 4, the morphology of the pancreas becomes indistinguishable from an adult mouse (Fig. 2c,d). As the pancreatic weight greatly augments from week 1 to week 4 (Fig. 1b) and the majority of the pancreatic volume consists of acinar cells, there is thus a substantial increase in acinar mass in the neonatal period. Ki67 staining shows massive proliferation of pancreatic acinar cells in this period (Fig. 2e–h). More than 40% of amylase + cells are positive for Ki67 at the age of 1 week. This is in sharp contrast with the proliferation level in adult acinar cells where less than 2% are Ki67 + ^31^. The proliferation level gradually decreases in the next few weeks after birth (Fig. 2f–h). Further, there were no clear indications for a regional difference in acinar proliferation in pancreatic tissue (border/centre of tissue, peri-insular/tele-insular) at all ages analysed.

Besides acinar cell proliferation, also acinar hypertrophy contributes to the increase in acinar cell mass as the acinar cell size increases by 48–80% between week 1 and week 4 in TAM and non-TAM-treated animals, respectively (Fig. 2i). TAM administration had no significant effect on proliferative activity of acinar cells but slightly reduced the increase in acinar cell size. Further, quantification of cellularity of acini revealed that the mean number of acinar nuclei per acinus is 9.3 ± 0.2 (n = 4) in 1-week old TAM-treated mice and that this number does not significantly differ at all ages analysed (Fig. 2j).

### Acinar cell number increases primarily by self-replication, as evidenced by lineage tracing

The important proliferative activity of neonatal acinar cells that was observed (see higher) does not exclude that part of the cells originated in the same period from a putative pancreas stem/progenitor cell. Therefore, we used the ElaCreERT R26-YFP mice to analyse whether acinar cell neogenesis from other cells than acinar cells may have occurred in the first 4 weeks of life. The lineage tracing method that we used to discriminate between progenitor-derived cells or the progeny of pre-existing cells, was based on the pulse-chase method, first described by Dor *et al*. for tracing beta cell neogenesis^32,33^. In our case, the acinar-specific elastase promoter rather than insulin was used. Mice received one TAM-injection on the day of birth. TAM will permanently induce YFP expression in acinar cells, which express elastase, and in their progeny during the chase (neonatal) period. However, if acinar cells would arise from non-acinar cells during the chase period, these will be unlabelled and cause a decrease in the labelling index of acinar cells. Such a dilution of the labelled acinar cells after the chase period, would prove the existence of acinar cell neogenesis from other cells than the originally elastase-positive acinar cells.

**Figure 3:**
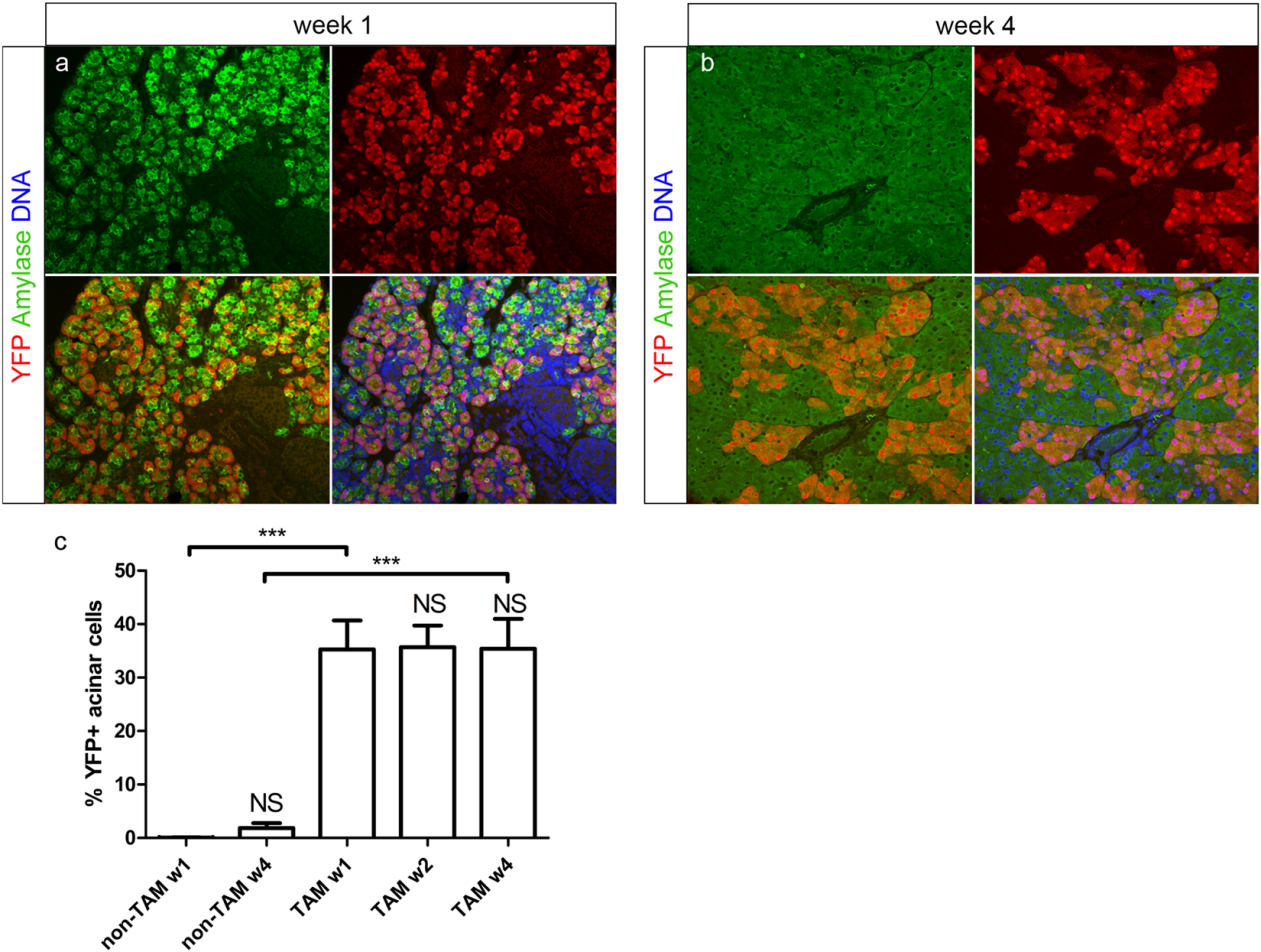
Tracing of acinar cell fate in ElaCreERT R26-YFP mice. Immunohistochemical staining for YFP and amylase at week 1 and 4 in TAM-treated mice (**a**,**b**). Shown are representative photomicrographs at original magnification of ×20. Percentage YFP positivity in acinar cells at indicated time points (**c**). Results are expressed as mean ± SEM. Data were analysed by 1-way ANOVA, followed by Bonferroni’s multiple comparison test (comparisons of interest: w1-w2, w2-w4, w1-w4; n1-w1, n4-w4, n1-n4). Results are considered statistically significant when P < 0.05. ***P < 0.001, NS: not significant. Detailed information about numbers of analysed mice, cells and area can be found in Table 1.

The pancreas of ElaCreERT R26-YFP was analysed for amylase, another acinar marker and YFP expression by immunohistochemistry. Most, if not all YFP+ cells expressed amylase in TAM-treated mice, indicating a high specificity of the tracer for acinar cells and confirming previous observations (Fig. 3a)^24^. The YFP-labelling efficiency of acinar cells, at 1-week of age was 35.3 ± 5.4% (n = 7) (Fig. 3a,c). This labelling index did not significantly change throughout the neonatal development, remaining 35.4 ± 5.6% by 4-weeks of age (n = 10) (p > 0.05) (Fig. 3b,c). This demonstrates that the acinar cell number primarily expands by proliferation of pre-existing acinar cells during the neonatal period and not by derivation from other cells like putative multipotent stem or progenitor cells or transdifferentiation. Further, our analysis showed that the %YFP+ duct cells accounted for 0.89 ± 0.34% of duct-lining cells at 4-weeks of age (n = 9), indicating that YFP labelling in these mice remains highly restricted to the acinar lineage.

### Duct cells do not contribute to the formation of acinar cells in the first month after birth

**Figure 4:**
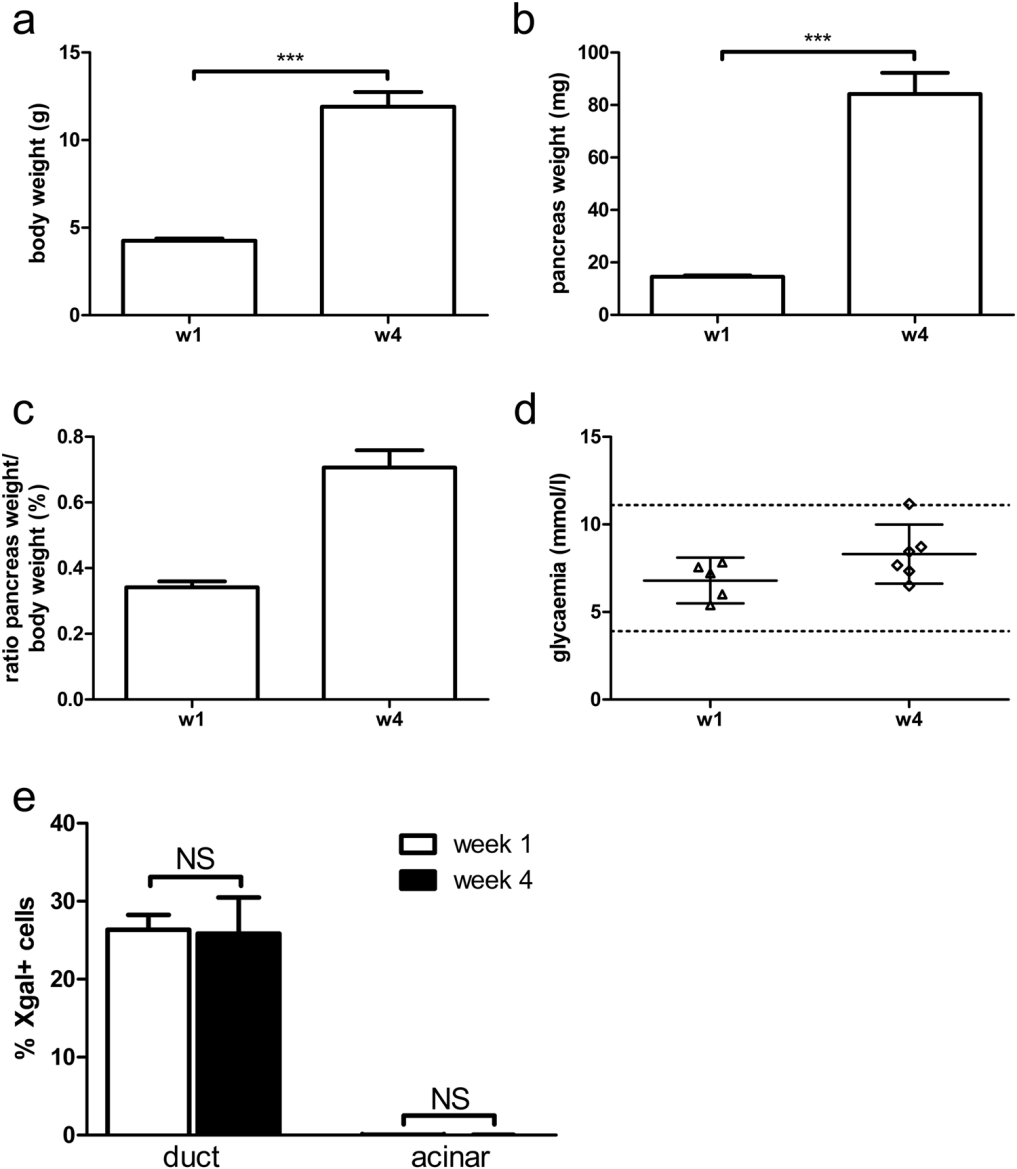
Follow-up and tracing of duct cell fate in Hnf1bCreERT R26R mice. Body weight (**a**), pancreas weight (**b**), ratio of pancreas weight over body weight (expressed as percentage) (**c**) and non-fasting glycaemia (**d**) of TAM-treated Hnf1CreERT R26R mice were measured at 1-week (n = 5) and 4-weeks of age (n = 6). Tracing of duct cell fate in TAM-treated Hnf1bCreERT R26R mice (**e**): quantification of X-gal + cells in ductal and acinar cell population at 1-week and 4-weeks of age. Data on glycaemia are expressed as mean ± 95% confidence intervals. All other results are expressed as mean ± SEM. (**a**,**b**,**e)** were analysed by unpaired two-tailed t-test. Results are considered statistically significant when P < 0.05. ***P < 0.001, NS: not significant. Detailed information about numbers of analysed mice, cells and area can be found in Table 1.

**Figure 5:**
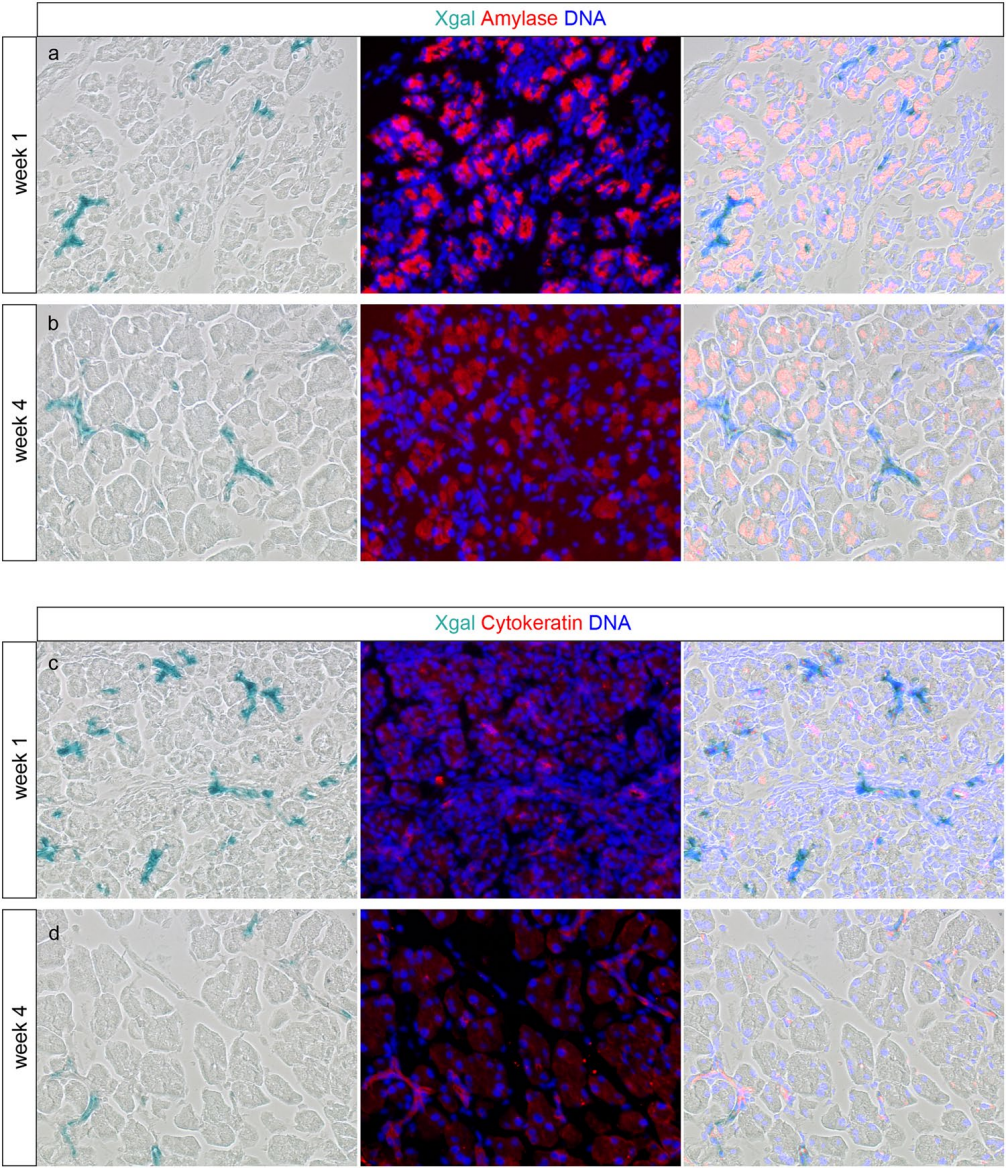
Specific duct-labelling and absence of acinar-labelling in Hnf1bCreERT R26-YFP. Pancreas from TAM-treated Hnf1bCreERT R26R mice were double stained for X-gal - amylase (**a,b**) and X-gal - cytokeratin (**c,d**) at 1-week (**a**–**c**) and 4-weeks (**b**–**d**) of age. At both time points X-gal remained restricted to cytokeratin + duct cells and was absent from amylase + acinar cells. Shown are representative photomicrographs at original magnification of ×20.

Duct cells have been regarded as progenitor cells of acinar cells, although several lineage tracing studies demonstrated the opposite. We previously showed that duct cells do not contribute to the formation of acinar cells after birth during physiological growth^29^. However, the period around weaning was not studied although it represents an important period for maturation of the pancreas as the diet changes from mother milk to solid food^34,35^. Therefore, we investigated the neonatal period using Hnf1bCreERT R26R mice to trace the fate of duct cells. Data on body weight, pancreas weight and glycaemia can be found in Fig. 4. With one TAM injection at the day of birth, 26.4 ± 1.9% of duct cells were labelled with beta-galactosidase as detected by enzyme histochemistry (n = 5) (Figs 4e and 5c). We previously showed extensive evidence that cells representing the differentiated pancreatic ductal epithelium including main, interlobular, intralobular, and centroacinar duct cells are being labelled randomly and solidly in these Hnf1bCreERT R26R mice^29^. The labelling specificity for duct cells in the pancreas is high as only 0.04 ± 0.01% of acinar cells are labelled at one-week of age (Figs 4e and 5a). If acinar cells arise from duct cells, increased labelling of acinar cells would be detected after the chase period. However, at 4-weeks of age, still a negligible amount of acinar cells is labelled (0.02 ± 0.02% X-gal+ acinar cells, p > 0.05), demonstrating that acinar cells do not arise from duct cells in the first month after birth (Figs 4e and 5b).

## Discussion

Our cell tracing results in ElaCreERT R26-YFP mice clearly demonstrate that acinar cells in the first 4 weeks of life do not derive from other cells than the acinar cells that were labelled immediately after birth. Since there was no dilution of labelled cells, acinar cells were derived from pre-existing acinar cells and there is no indication that putative stem or progenitor cells would have contributed to the acinar cell mass. Our observations on elevated Ki67-labelling confirms that the massive expansion of the acinar cell population occurs by self-division.

This conclusion is in sharp contrast with two studies that reported derivation of acinar cells in the neonatal period from duct cells, one making use of human carbonic anhydrase-II^27^ and the other of Sox9^26^ to trace duct cells in the mouse. However, our results with Hnf1bCreERT R26R mice contradict their observations and are in line with two other duct-tracing studies that found no evidence for a duct-to-acinar cell contribution in neonatal and adult mice^28,29^. Another tracing study using the acinar marker Ptf1a confirmed that in adult mice acinar cells do not derive from other cells^11^. A similar conclusion was drawn by a study tracing both acinar cells and duct cells and reporting no spontaneous cell conversion postnatally^17^.

Several explanations exist for the discrepancy between these results and those from the two studies mentioned^26,27^. The Inada study made use of the human CAII-promoter to trace duct cells but it has been previously reported that this transgene may be inappropriately expressed in mouse tissues^36^. Furthermore, to study neonatal development they used a Cre model which was not inducible (CAII-Cre), and thus did not allow pulse-chasing labelling like in the other studies. Cre models, unlike CreERT, provide continuously active labelling and thereby Cre activation in other cell types than CAII+ cells cannot be excluded^27^. As to the Sox9 tracer study of Furuyama *et al*., their results were contradicted by those from Kopp *et al*. who also used a Sox9-driver but did not find a contribution of Sox9+ duct cells to the acinar population. This discrepancy could be explained by the fact that Kopp *et al*. used BAC Sox9-CreERT mice whereas in Furuyama’s mice the IRES-CreERT2 cassette was inserted in the 3′UTR of the endogenous Sox9 locus^26,37^. It has been suggested that the altered structure of the Sox9 locus as a result of this insertion can cause a reduction of Sox9 expression^38^. At postnatal day 1, Sox9 expression was not yet altered but later on, Sox9 expression was significantly reduced in the Sox9IRESCreERT2 mice. Sox9 dosage has been shown to play an important role in ductal plasticity and reduced Sox9 expression may allow duct cells to erroneously differentiate into acinar cells in these Sox9IRESCreERT2 mice^38^. This problem is not present in the BAC transgenic mice which therefore allow a more faithful expression of Sox9 and more reliable tracing results^28^.

Our observation that the mean number of acinar nuclei per acinus remains constant throughout the first 4 weeks of life in combination with a very high proliferative activity of acinar cells in this period implies that acini form new acini by budding off from expanding acini. A classical view of pancreatic architecture has been that of a bunch of grapes and this view has led to the interpretation by histologists that acini might originate as structures budding off from ducts. However, studies making use of corrosion casts of pancreatic tissue revealed that postnatal acini can also take branching shapes^39^. This observation could reflect the way in which acinar tissue actually expands as a consequence of cell division rather than neogenesis from ducts.

Studying cell turnover under physiological conditions is important since this knowledge is needed to be able to explore and compare with pathological conditions. Our study adds up to the growing number of studies which show that in homeostatic conditions, postnatal pancreatic epithelial cells are self-renewing and that their populations do not depend on (multipotent) stem cells^11,17,24,28,29,32,40,41^. Our present study focused on a period of life that has been less well investigated but that represents an important period of major expansion for the exocrine pancreas, namely the first four weeks after birth. According to our observations, the thirteen to fourteen-fold increase in exocrine cell mass in this period originates from a combination of mitotic division and hypertrophy of the acinar cells that were present at the day of birth.

Under other pathological or experimentally induced conditions, acinar cells show a remarkable cell plasticity and are able to form other cell types including duct cells and beta cells^6,7,14–20,24,25,42^.

This combined knowledge opens perspectives for new treatments for major pancreas pathologies such as pancreas cancer and diabetes.

## Methods

### Animals and experimentation

The following transgenic mouse strains were used: ElastaseCreERT Rosa26-Lox-STOP-Lox-EYFP (ElaCreERT R26-YFP) (Doris Stoffers/Patrick Jacquemin^24^), Hnf1bCreERT R26R (Jorge Ferrer^29^). All animal experiments were conducted as approved by our institutional Ethical Committee of Animal Experimentation, and were in accordance with the European guidelines for animal experimentation and with national regulations.

Mice were mated, males were removed from the cage before delivery and at the day of birth, postnatal day 0 (P0), pups were injected intraperitoneally with freshly prepared 0.5 mg tamoxifen (TAM) (Sigma-Aldrich, Diegem, Belgium). TAM was dissolved at 20 mg/ml, with aid of sonication in 0.9% NaCl and 10% EtOH and kept on ice. Mice were kept on standard chow (A03, Safe Diets, Augy, France). Pups remained with mother until analysis. Non-fasting glycaemia was measured at endpoint using GlucoMen LX plus sensors (A. Menarini Diagnostics, Firenze, Italy).

### Immunohistochemistry and microscopy

Paraffin sections and cryosections were prepared as described in^43^. 5-bromo-4-chloro-3-indolyl-beta-D-galactopyranoside (X-gal)-staining was performed on cryosections as described in^29^, followed by immunohistochemical staining as described below. For immunohistochemical staining we used the indirect method with fluorochrome-labelled secondary antibodies. Primary antibodies were anti-amylase (rabbit, 1/500, Sigma-Aldrich), anti-Ki67 (rat, 1/5000, eBioscience), anti-GFP (GTX26658, goat, 1/100, Bioconnect (Huissen, The Netherlands)), anti-cytokeratin (WSS, rabbit, 1/2000, Dako). For paraffin sections, heat-mediated antigen retrieval was used for anti-GFP and for anti-Ki67 using target retrieval solution (S1699, Dako) for 20 minutes at 99 °C and 20 minutes cooling down at bench. Enzyme-mediated antigen retrieval was used for anti-cytokeratin by incubation with 15ug/ml proteinase K (Novocastra) in a buffer containing 100 mM Tris pH8 and 50 mM EDTA pH8 for 18 min at 37 °C. Secondary antibodies coupled to fluoresceinisothiocyanate, tetramethyl rhodamine isothiocyanate, cyanine 3 and AlexaFluor 594 were used (all from Jackson ImmunoResearch Laboratories, West Grove, PA., USA). Nuclei were labelled by Hoechst (5 µg/ml) (bisBenzimide H 33342 trihydrochloride, Sigma-Aldrich). Sections were mounted using Vectashield mounting medium (Vector Laboratories, Burlingame, CA, USA).

Microscopic images were acquired with a Nikon Eclipse 90i microscope using NIS Elements AR 3.10 software or with a multiphoton confocal microscope Zeiss LSM710 using ZEN 2009 6.0 software. Microscopic images (20x magnification) were taken from different tissue regions (border/centre of tissue, peri-insular/tele-insular) to ensure a representation of all regions, and cell counts were performed on at least 6–9 images from at least 3 non-consecutive sections.

Morphometric analysis was performed on paraffin sections stained for amylase. Acinar cell size was calculated by dividing the amylase + cell area by the number of nuclei of amylase + cells in that area.

Cellularity of acini was determined on paraffin sections stained for amylase. The number of acinar nuclei was counted per acinus. To exclude acini that were cut out of their centre, only acini with a central lumen or centroacinar cell were included.

### Statistical analysis

Data on glycaemia are expressed as mean ± 95% confidence intervals. All other results are expressed as mean ± SEM. All data were analysed by unpaired two-tailed t-test (Fig. 4a,b,e) or 1-way ANOVA (Figs 1a,b,c, 2h,i,j and 3c), followed by Bonferroni’s multiple comparison test (comparisons of interest: w1-w2, w2-w4, w1-w4; n1-w1, n4-w4, n1-n4. For Fig. 1c: n1-w1, n4-w4); after normality testing. Results are considered statistically significant when P < 0.05. All analyses were performed using GraphPad Prism version 5.01 for Windows. * or °: P < 0.05, ** or °°: P < 0.01, *** or °°° P < 0.001. Detailed information about numbers of analysed mice, cells and area can be found in Table 1.

**Table 1:** Number of mice and cells per analysis.

	non-TAM w1		non-TAM w4		TAM w1		TAM w2		TAM w4	
	mice	cells	mice	cells	mice	cells	mice	cells	mice	cells
%Ki67 + acinar cells	4	45231	4	33655	4	49704	4	52783	4	41435
acinar cell size	3	24557	7	40883	7	39054	4	37343	10	54609
ElaCreERT mice %YFP + acinar cells	9	54557	11	60883	7	47368	4	37343	10	54609
ElaCreERT mice %YFP + duct cells									9	6656
Hnf1bCreERT mice %X-gal + duct cells					5	8583			4	8303
Hnf1bCreERT mice %X-gal + acinar cells					5	67597			4	31351
Cellularity acini	4	240 acini	4	240 acini	4	240 acini	4	240 acini	4	240 acini

### Data availability statement

All relevant data from this study are included in this article.
